# A Case Report on an Intricate Forensic Dilemma: Precipitate Labor or Neonaticide?

**DOI:** 10.7759/cureus.89092

**Published:** 2025-07-30

**Authors:** Santanu Das, Utpal Tripura, Gambhir Singh, Ninad V Nagrale

**Affiliations:** 1 Forensic Medicine, Agartala Government Medical College, Agartala, IND; 2 Forensic Medicine, All India Institute of Medical Sciences, Kalyani, Kalyani, IND

**Keywords:** feticide, neonatal death, neonaticide, precipitate labor, psychology

## Abstract

Precipitate labor and neonaticide can present with overlapping features, often posing significant challenges in forensic interpretation. Differentiating between these possibilities is essential for reaching accurate medicolegal conclusions. This case report examines the forensic complexities involved in distinguishing precipitate labor from neonaticide. A father brought a deceased neonate to the hospital, alleging that the mother had fatally slit the infant’s throat. In contrast, the mother claimed the injury occurred accidentally during an unexpected bathroom delivery. She stated that while attempting to cut an umbilical cord looped around the baby’s neck with a blade, she unintentionally caused the fatal wound. Autopsy findings revealed a full-term male neonate with multiple incised wounds on the neck, blunt trauma to the scalp, and bilateral subdural and subarachnoid hemorrhages. The combination of sharp and blunt force injuries, along with conflicting parental accounts, raised concerns about the true circumstances surrounding the death. Further complicating the case were the father’s delayed reporting and his claim of being unaware of the pregnancy. This report highlights the critical need for a comprehensive forensic investigation, including careful correlation of injuries with reported events and consideration of medical, psychological, and social factors in suspected cases of neonaticide.

## Introduction

Precipitate labor, defined as the expulsion of a fetus within less than three hours of the onset of contractions or abdominal pain, occurs in approximately 2% of deliveries, most commonly in multiparous women. It poses significant risks to the fetus, including intracranial hemorrhage and trauma resulting from rapid delivery, particularly when it occurs in a standing position [1].

Neonaticide - the intentional killing of a child within the first four weeks of life - has an incidence of 2.4 to 7.0 per 100,000 live births in industrialized countries, with the highest risk occurring on the first day of life and a greater prevalence among female neonates [2-6]. Common methods of neonaticide include direct injury or neglect [7].

Distinguishing neonatal death due to precipitate labor from neonaticide presents considerable forensic challenges, as injuries sustained during a rapid, uncontrolled delivery may resemble those caused by deliberate harm. This article presents a complex case involving conflicting parental statements, detailed autopsy findings, and the relatively rare occurrence of male neonaticide. The case underscores the difficulty of determining the exact cause and manner of death and highlights the importance of thorough forensic evaluation to differentiate between accidental death and intentional killing.

## Case presentation

A male individual (the father of the deceased), employed as a driver, brought a deceased neonate to the emergency department at approximately 6:20 AM. He reported that the mother had fatally injured the neonate’s throat at around 12:30 AM and claimed complete unawareness of her pregnancy. The mother, a multiparous woman with two previous living children, provided a different account. She stated that she had delivered the neonate unexpectedly in the bathroom, was unaware of the sudden fetal descent, and accidentally injured the neck while attempting to cut an umbilical cord looped around the neonate’s neck using a safety razor blade.

External examination

**Figure 1:**
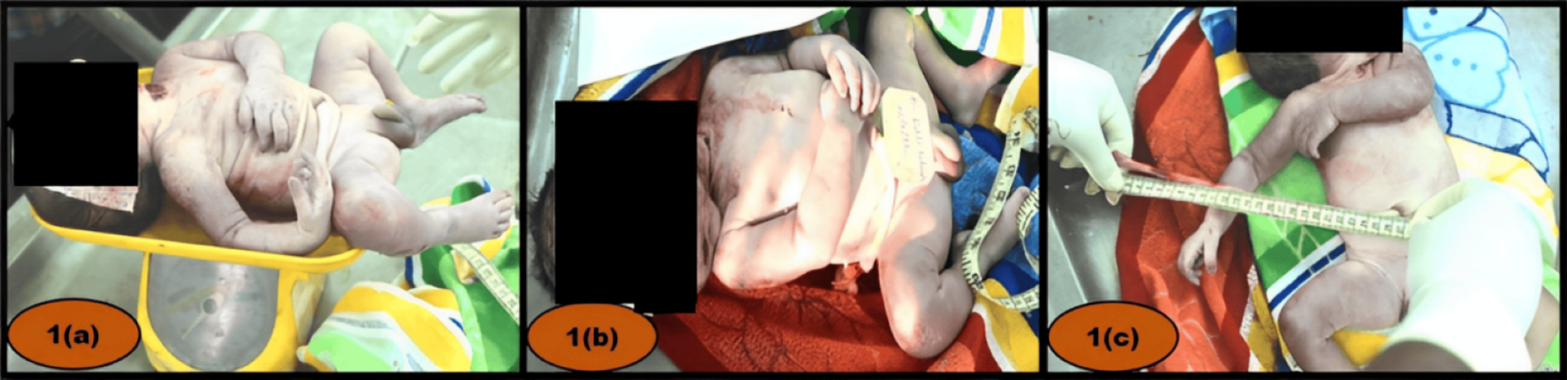
(a) Weight measurement of the neonate prior to the postmortem examination. (b) Full-term neonate with visible blood stains. (c) Umbilical cord showing a cut end positioned away from the neonate.

A mature, full-term male infant weighing 2.8 kg was received (Figure 1a), with a crown-heel length of 52 cm. Both eyes and the mouth were closed. Dried blood stains were observed over the neck and anterior chest (Figure 1b). Meconium was present in and around the anal canal. The nail beds were cyanosed, and the nails extended beyond the nail beds. Both testes were completely descended into the scrotum. A brown-colored identification tag was tied to the umbilical stump. The umbilical cord stump measured 27 cm in length and had a clean-cut distal end with blood present at the margins (Figure 1c).

Multiple postmortem ant bite marks measuring 0.1 cm × 0.1 cm to 0.8 cm × 0.1 cm were noted on the outer aspect of the left cheek, extending to the left temple over an area of 6 cm × 2 cm, located approximately 2 cm anterior to the left ear.

External and internal injuries

Antemortem Findings

**Figure 2:**
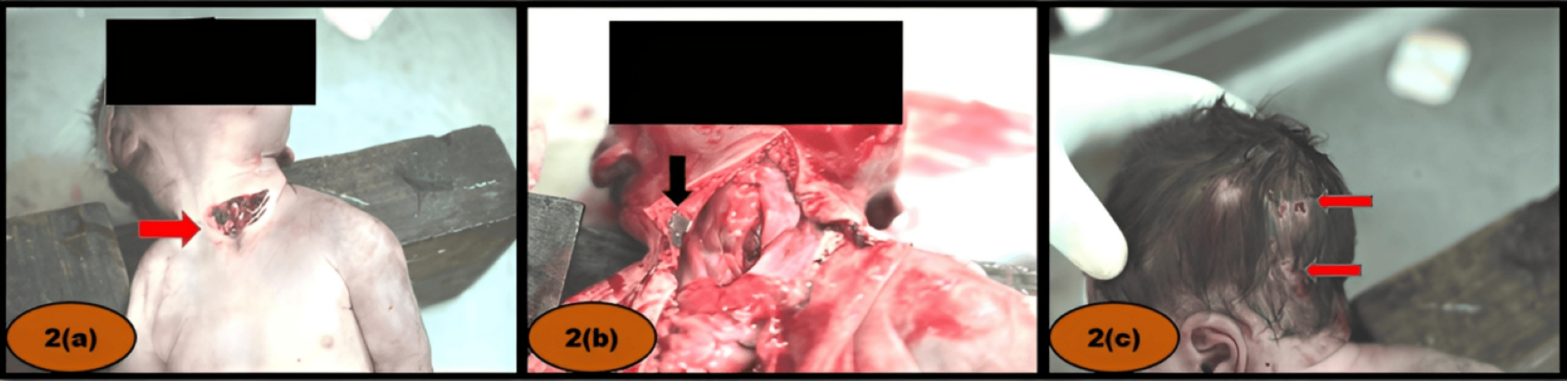
(a) Incised wound on the anterior aspect of the neck. (b) Neck muscles without evidence of contusion. (c) Abrasion on the left side and back of the head.

Primary neck injury: A spindle-shaped incised wound measuring 4 cm × 2 cm was present, extending into the tracheal lumen. It was positioned horizontally across the midline of the anterior neck. The upper margin was located 5 cm below the chin, and the lower margin lay just above the suprasternal notch. The underlying superficial vessels, platysma, and trachea were incised (Figure 2a).

Forensic significance: The depth and location of the wound suggested the use of significant force. The absence of bruising in the strap muscles indicated minimal or no struggle and no attempt to compress the neck, as would be expected in a case of throttling.

Secondary neck injuries: Two additional spindle-shaped incised wounds were noted. The first measured 1 cm × 0.1 cm and was skin deep, positioned horizontally below the left lower margin of injury no. 1. The second measured 2 cm × 0.1 cm and was also skin deep, positioned horizontally below the lower margin of injury no. 2.

**Figure 3:**
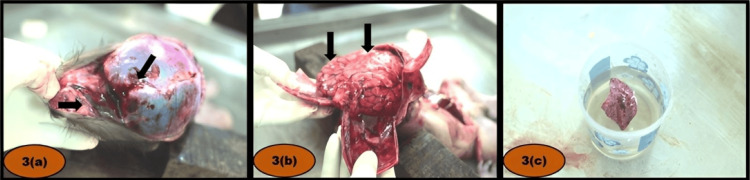
(a) Extravasation of blood within the layers of the scalp. (b) Diffuse subarachnoid hemorrhage. (c) Positive flotation test.

Internal examination findings: No bruising was observed in the strap muscles (Figure 2b). Blood clots were found within the airways, extending to the terminal bronchioles. Extravasation of blood was present within the scalp layers over the bilateral parieto-temporo-occipital regions. A subdural hemorrhage was noted over the right occipital lobe, along with subarachnoid hemorrhage over the bilateral cerebral hemispheres (Figure 3a, 3b).

Head injury: An abrasion measuring 5 cm × 2 cm was observed on the left side of the posterior head, located 4 cm from the midline and just below the left parietal eminence (Figure 2c).

Postmortem Findings

Multiple ant bite marks were present on the left cheek and temple region.

## Discussion

The cause of death was attributed to the combined effects of head and neck injuries. The head injury was consistent with impact from a hard, blunt object, while the neck injury exhibited characteristics indicative of a sharp cutting instrument. However, the exact sequence of these injuries could not be determined, resulting in an ambiguous classification regarding the manner of death. Evidence of live birth was established based on normal skin coloration, a clean-cut umbilical cord (although lacking a ligature) located away from the fetal end (Figure 1c), absence of maceration, presence of caput succedaneum, and a positive hydrostatic test (Figure 3c).

Forensic significance: The clean-cut umbilical cord and positive hydrostatic test confirmed live birth, effectively ruling out stillbirth. Literature on neonaticide frequently documents methods such as strangulation, smothering, stabbing, drowning (e.g., in bathtubs or toilets), or abandonment [5]. Neonaticide is more commonly observed in female neonates and is often associated with unwanted pregnancies, denial of pregnancy, congenital anomalies, marital discord, extramarital affairs, maternal mental illness, or unfavorable social and economic circumstances, with higher prevalence among unmarried mothers [8]. In some regions, particularly in parts of Asia, neonaticide of female infants may be linked to gender-based discrimination and perceptions of socioeconomic burden [9].

This case deviated from typical patterns, as the victim was male [9] and the underlying motives remained unclear. While low socioeconomic status was reported, the broader family context did not align with common predictors of neonaticide. The mother’s account - claiming that the neck injury occurred accidentally during precipitate labor - showed inconsistencies when compared with the observed injury pattern, particularly the horizontal orientation of the primary neck wound. Additionally, the father’s claim of being unaware of the pregnancy appeared implausible given their cohabitation and the likely visibility of an advanced pregnancy.

These discrepancies posed significant challenges in reconstructing the sequence of events and determining intent. The presence of both blunt and sharp force injuries suggested a complex mechanism of trauma that was inconsistent with the explanations provided. Several investigative questions remained unresolved, including (a) What factors contributed to the delayed hospital presentation? and (b) What were the circumstances leading to the retention of the neonate at the scene?

This case underscores the forensic complexities involved in neonatal death investigations. Further recommended steps include histopathological examination of the lungs to corroborate evidence of live birth (complementing the hydrostatic test), DNA analysis to confirm paternity, investigation of the delivery site for supporting circumstantial evidence, and psychological assessment of both parents to better elucidate the sequence of events.

## Conclusions

Although this case lacks clear indicators of intent commonly associated with neonaticide, the available evidence warrants cautious interpretation, particularly in light of the reported circumstances and injury patterns. The findings highlight the need for comprehensive forensic investigation, including crime scene evaluation, as distinguishing between accidental and intentional factors in neonatal deaths requires careful consideration of social, psychological, and pathological factors. Delivery site inspection and psychological evaluation of both parents are essential for providing further insight into the case.
